# Electrically driven, highly efficient three-dimensional GaN-based light emitting diodes fabricated by self-aligned twofold epitaxial lateral overgrowth

**DOI:** 10.1038/s41598-017-10086-7

**Published:** 2017-08-29

**Authors:** Yang-Seok Yoo, Hyun Gyu Song, Min-Ho Jang, Sang-Won Lee, Yong-Hoon Cho

**Affiliations:** Department of Physics, Korea Advanced Institute of Science and Technology, Daejeon, 34141 Republic of Korea

## Abstract

Improvements in the overall efficiency and significant reduction in the efficiency droop are observed in three-dimensional (3D) GaN truncated pyramid structures fabricated with air void and a SiO_2_ layer. This 3D structure was fabricated using a self-aligned twofold epitaxial lateral overgrowth technique, which improved both the internal quantum efficiency and the light extraction efficiency. In addition, a reduced leakage current was observed due to the effective suppression of threading dislocations. While this study focuses primarily on the blue emission wavelength region, this approach can also be applied to overcome the efficiency degradation problem in the ultraviolet, green, and red emission regions.

## Introduction

In recent years, *c*-plane GaN-based light emitting diodes (LEDs) have been used in diverse mass-market applications including general illumination, full-color visual displays, traffic signals, and backlights for liquid-crystal display panels. However, the LED efficiency is limited by point defect and high threading dislocations (TDs) caused by lattice mismatching and differences in the thermal expansion coefficients between the substrate of the LED and the layers grown on the substrate^1, 2^, point defect for non-radiative center^3, 4^, the large internal electric field existing within the active region^5^, carrier overflow due to the asymmetry of the electron-hole concentration or polarization, Auger recombination, junction heating effect, carrier delocalization effect, current crowding effect, and density activated defect recombination etc. Also, total internal reflection resulting from different refractive indices in the GaN layer (*n*_r_ ~ 2.5) and the surrounding air (*n*_r_ ~ 1.0) suppressed the LED efficiency^6^.

A number of useful growth techniques have been proposed by several groups in order to reduce TDs, including the epitaxial lateral overgrowth (ELOG)^7^, pendeo-epitaxy (PE)^8^, maskless PE^9^, cantilever epitaxy^10^, facet-controlled epitaxial lateral overgrowth (FACELOG)^11^, and two-step ELOG^12^. In addition, various methods such as surface texturing of *p*-GaN layers^13^, insertion of a photonic crystal structure^14^, and flip chip^15^ or vertical LED structures^16^ have been utilized in order to improve the light extraction efficiency (LEE) of these systems. However, these growth techniques require complex growth conditions such as change of growth temperature and V/III ratio etc. In addition, complex processes are often required for the use of photonic crystals and the fabrication of flip chip or vertical LEDs. Most previous studies have also focused solely on either reduction of the TDs or increasing the LEE of LEDs, but not both.

Various nano- and micro-scale structures having semi- or non-polar facets have been reported as having many advantages for enhancing the internal quantum efficiency (IQE) and LEE of these systems^17, 18^. Such structures can be fabricated by “bottom-up” or “top-down” approaches. Bottom-up techniques include self-assembled^19^, catalyst-driven^20^, and selective area growth^21^ methods, while some examples of top-down techniques include dry or chemical etching methods^22^. Despite the successful formation of some nano- and micro-scale structures using these methods, the uniformity and reproducibility of some bottom-up structures (such as vapor-liquid-solid methods using metal catalysts) are low, and contamination issues arise due to the use of a metal catalyst^23^. Since the fabrication is relatively easier in the top-down case when compared with that in the bottom-up approach and allows for an effective size control, the top-down approach has been widely utilized for the fabrication of nano- and micro-scale structures. However, the quantum efficiency of samples fabricated in this manner is relatively lower than that grown by in a bottom-up approach, due to significant physical etching damage^24^. In addition, the TDs in samples fabricated in a top-down approach commonly extend in the *c*-direction from the substrate because of large lattice mismatches between the substrate and the layers grown on the substrate. This is in contrast with the case of the bottom-up approach, in which lateral bending of some dislocations from the initial propagation direction of TDs occurs^25^. Thus, special care is required to increase the efficiency of samples fabricated using top-down approaches.

Here, we propose a self-aligned twofold epitaxial lateral overgrowth (STELOG) technique to fabricate highly efficient three-dimensional (3D) LED structures. Generally, other growth techniques use two or more steps of growth conditions to achieve lateral growth. For instance, the actual process of 2-STEP ELOG is very complicated. However, our proposed method (i.e., 1-STEP, self-aligned twofold ELOG) with only one-step regrowth procedure does not need to change the growth conditions since it will merge spontaneously between adjacent pillars. In this technique, an air void and a SiO_2_ layer are introduced during the regrowth of GaN pillar structures (referred to as AS-LED), which dramatically decreases the number of TDs, the stress of the epilayer, and the effect of total internal reflection, improving the overall efficiency and reducing the efficiency droop. These characteristics were also compared to structures having only air voids (referred to as A-LED) and conventional planar LEDs (referred to as P-LED). In addition, the leakage current in AS-LED was largely reduced compared to that of other samples (Supplementary Section IV). Finally, the emission wavelength for AS-LED was much longer than that of the other samples, because of the increased lattice pulling effect from stress relaxation (Supplementary Section V).

## Results

### Self-aligned twofold epitaxial lateral overgrowth

**Figure 1:**
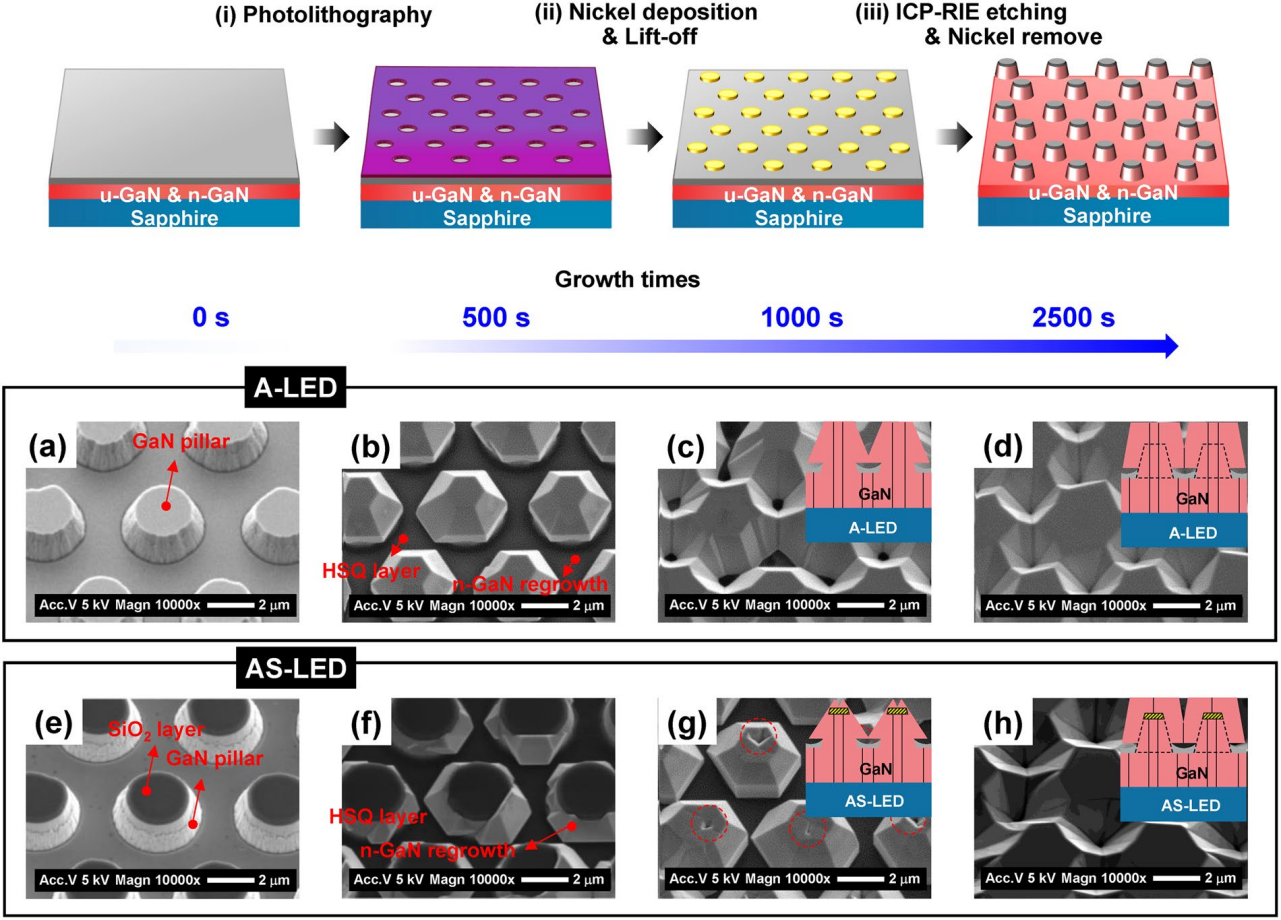
Schematics of the GaN pillar structure fabrication, and investigations of different growth modes for A-LED and AS-LED based on growth times. (**a**–**h**) Top-view SEM images for A-LED and AS-LED at different growth times (*t* = 0, 500, 1000, 2500 s). The SiO_2_ layer is observed at the top region of AS-LED (**e**). An HSQ layer was added to avoid the regrowth of *n*-GaN and MQWs between GaN pillar structures. Although the *n*-GaN layer in A-LED is completely regrown on the surface of the GaN pillar structure, the *n*-GaN regrowth in the AS-LED is only observed around the tapered GaN pillar structure except for the *c*-plane area (**b**,**f**). The coalescent region in the AS-LED was observed in the dotted circle region, unlike the A-LED (**c**,**g**). By increasing the growth time even further, we observed nearly identical flat *c*-plane surfaces in both samples (**d**,**f**).

The LED samples used in this study were fabricated through the regrowth of GaN pillar structures made by a top-down approach as follows. First, a 300-nm-thick SiO_2_ layer was deposited on an *n*-GaN layer on a sapphire substrate by plasma-enhanced chemical vapor deposition. Then, the SiO_2_-deposited GaN pillar structures can be fabricated through a series of processes including photolithography, nickel deposition, lift-off, induced coupled plasma-reactive ion etching, and removal of the nickel mask (Fig. 1(i)–(iii), Supplementary Section I). Note that the SiO_2_ layer was not removed in order to suppress the extension of TDs to QWs in the *c*-direction from the substrate for the AS-LED sample; the GaN pillar structures without this SiO_2_ layer were also prepared by removing the SiO_2_ layer (for A-LED) for comparison. Figure 1a and e shows top-view scanning electron microscopy (SEM) images for the GaN pillar templates without and with a SiO_2_ layer for A-LED and AS-LED, respectively, prior to regrowth. Note that the SiO_2_ layer is seen only in the top region of AS-LED (Fig. 1e). In order to prevent the regrowth of *n*-GaN and MQWs between GaN pillar structures in both samples, a hydrogen silsesquioxane (HSQ) layer was added by spin coating between the GaN pillar structures, as shown in Fig. 1b,f.

Figure 1b–d and f–h show SEM images of A-LED and AS-LED structures at different growth times (*t*). The *n*-GaN regrowth was observed at *t* = 500, 1000, and 2500 s, and distinct differences of the growth modes were observed between the two samples. At *t* = 500 s, an *n*-GaN layer in A-LED was completely regrown on the entire surface of the GaN pillar structure (Fig. 1b), while the *n*-GaN regrowth in AS-LED was only observed around the GaN pillar structure except for the *c*-plane area with the SiO_2_ layer (Fig. 1f). At *t* = 1000 s, a coalescent region was observed in AS-LED within the dotted circle region marked in Fig. 1g, unlike A-LED (Fig. 1c). We note that the coalescent region is a strong evidence of ELOG on top of the SiO_2_ layer. With time, the coalescent region observed in AS-LED decreased in size, and we finally observed the same flat *c*-plane surface at *t* = 2500 s in both samples (Fig. 1d and h). It is worth noting that by using only a one-step regrowth process, twofold epitaxial lateral overgrowth can be achieved in a self-aligned fashion for AS-LED structures. A clear difference in the cross-sectional structures between A-LED and AS-LED was confirmed through cross-sectional SEM imaging (Supplementary Section II).

### Characterization of structure and cathodoluminescence

**Figure 2:**
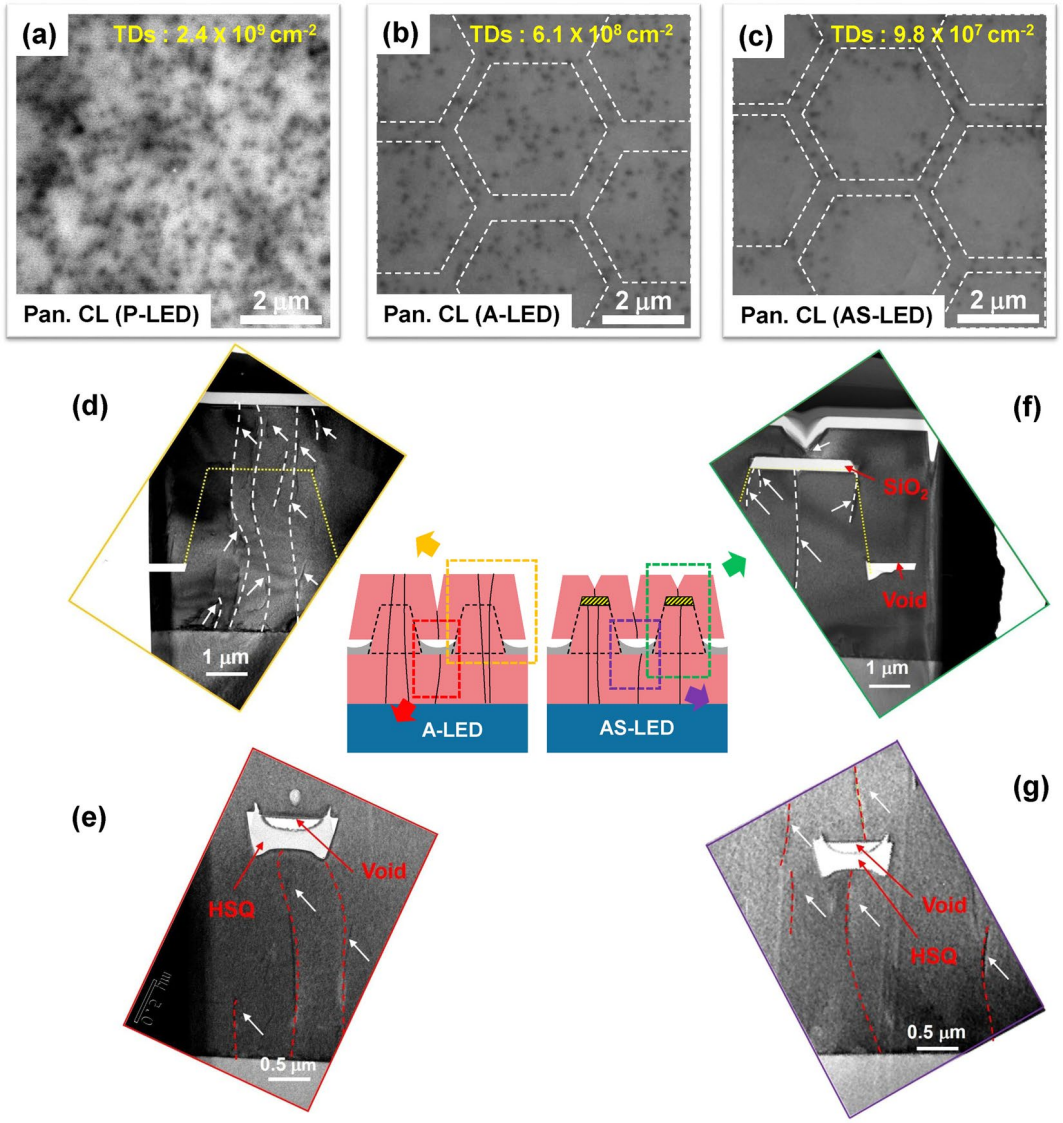
Differences in material quality between the samples: (**a**–**c**) panchromatic CL images, and (**d**–**g**) TEM images for P-LED, A-LED, and AS-LED. The hexagonal white dotted lines in the CL images indicate 3D structures.

We performed various experiments to clarify the differences in material quality between samples. Figure 2a–c shows panchromatic cathodoluminescence (CL) images of P-LED, A-LED, and AS-LED samples, respectively. The dark spot region in the CL images indicates non-radiative recombination centers and the dotted hexagonal lines indicate the *c*-plane area of the truncated GaN pyramids. The dark spot densities were found to be 2.4 × 10^9^, 6.1 × 10^8^, and 9.8 × 10^7^ cm^−2^ for P-LED, A-LED, and AS-LED, respectively, showing effective suppression of TDs by air voids and SiO_2_ layers. These findings are coincident with the transmission electron microscopy (TEM) results shown in Fig. 2d–g. Based on the TEM data, we found that TDs induced between the substrate and the layer grown on the substrate were effectively blocked by the HSQ layer and the SiO_2_ layer. The new threading dislocations were partially observed in merged region of *n*-GaN regrown on the SiO_2_ layer and the HSQ layer as shown in merged region of general ELOG after GaN regrowth. However, we expect that the bundle of TDs generated directly from the substrate will be much more dominant than the new threading dislocations appeared at the merged region. The differences in material quality between the samples were also confirmed by *ω*-scan rocking curves from high resolution X-ray diffraction (HRXRD). The full width at half maximum (FWHM) of the symmetry (002) *ω*-scan curves for all samples show similar values, although the FWHM value for P-LED is slightly larger than that of the others. On the other hand, FWHM of the asymmetry (102) *ω*-scan curve for AS-LED shows the smallest value among the samples (Supplementary Section III). These results show that the material quality in AS-LED was much improved compared to that in P-LED and A-LED.

**Figure 3:**
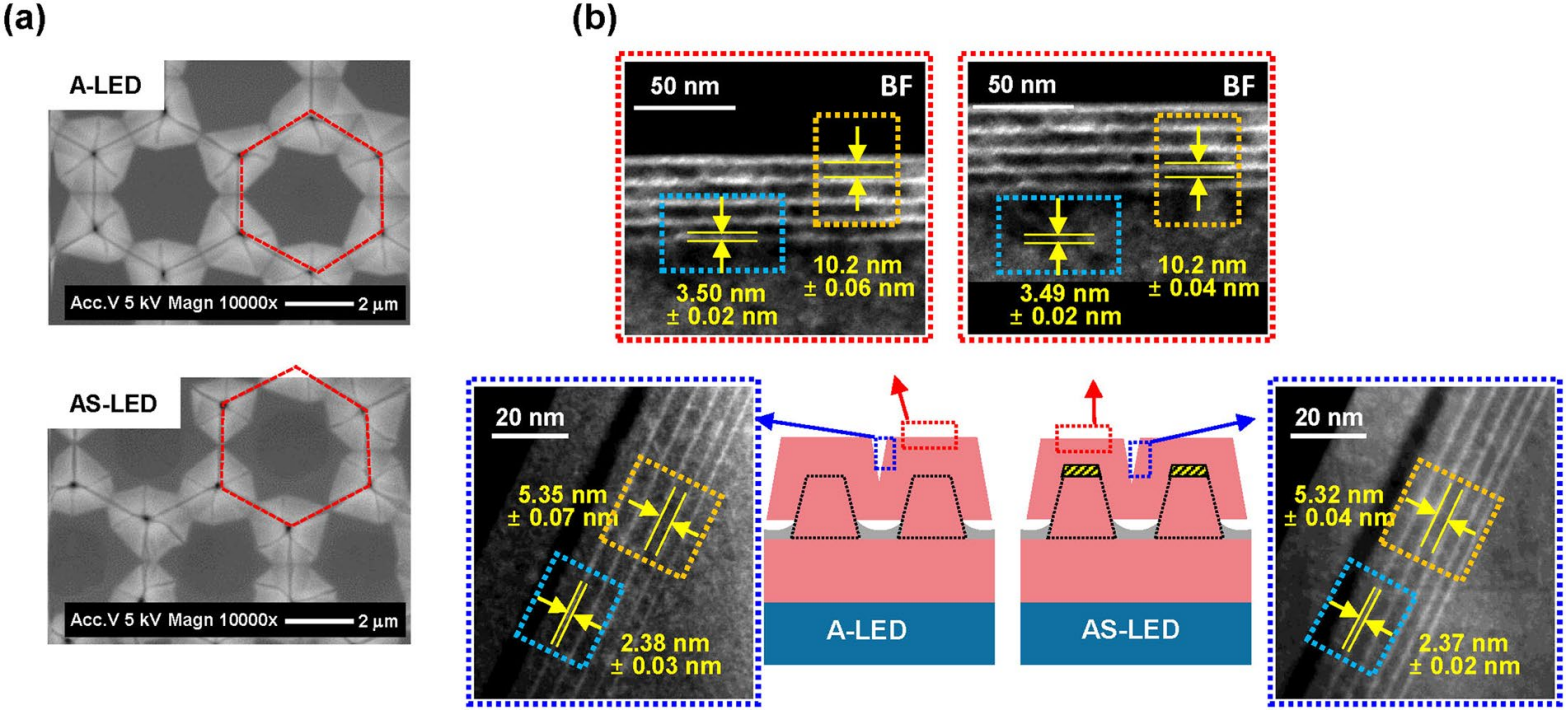
TEM measurements of the effective active volume: (**a**) Top-view SEM image and (**b**) TEM images of QWs in semi-polar and polar facets for the A-LED and AS-LED. The hexagonal red dotted line in (**a**) indicates the unit cell area.

In general, the InGaN/GaN QW core/shell structure is known to decrease the effective carrier density, due to having a larger active area than that of planar InGaN/GaN QW structures made under the same carrier pumping conditions^16^. In this study, five InGaN/GaN QWs were grown for P-LED, A-LED, and AS-LED samples. Figure 3 compares the differences of the unit cell area and effective active volume for three samples. The unit cell area (dotted line) and effective active volume of A-LED and AS-LED were calculated from the top-view SEM images (Fig. 3a) and measurements of the well thickness by TEM (Fig. 3b), respectively, as listed in Table 1. We confirmed that the effective active volume for A-LED and AS-LED increased by approximately 30% compared to that of P-LED. Thus, we expect that the effective carrier density in QWs for A-LED and AS-LED will be much reduced than that of P-LED under the same pumping conditions, due to the increased relative effective active volume compared to that of P-LED.

**Table 1:** Calculations of unit cell area and effective active volume.

Sample	Unit cell area	Effective active volume	Well thickness	
P-LED	8.89 μm^2^	0.16 μm^3^	**~**3.5 nm	
A-LED, AS-LED	~13.74 μm^2^	~0.21 μm^3^	Semipolar **~**2.4 nm	*c*-plane **~**3.5 nm
	~54.5% ↑	~31.2% ↑		

### Light extraction efficiency

**Figure 4:**
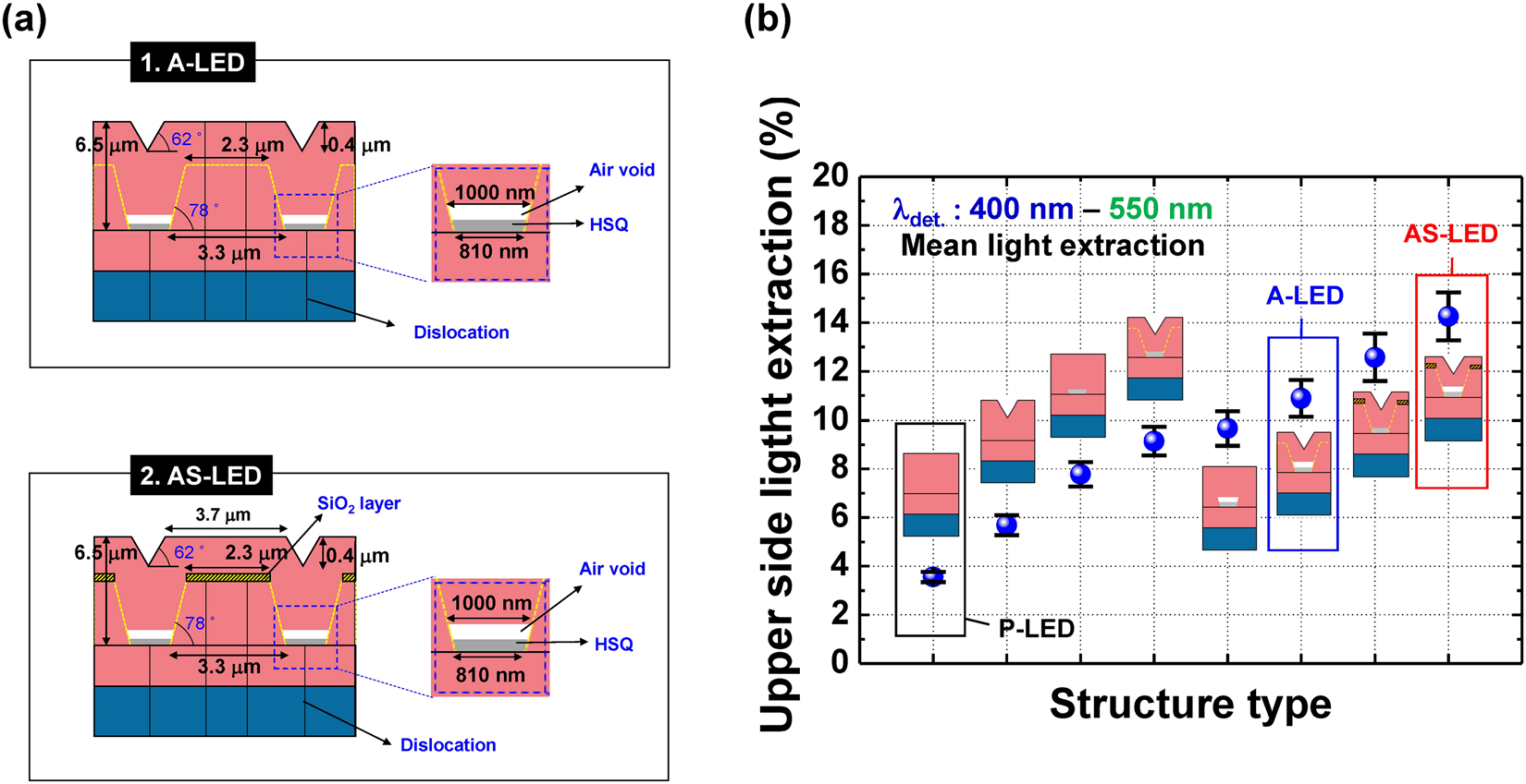
FDTD simulation results for light extraction of different sample structures: (**a**) Side view of the simulated sample structure for the A-LED and AS-LED. (**b**) Upper side light extraction efficiency for various simulated structures; *λ*_det._ was set at 400–550 nm, based on the EL data of the sample.

Finite-difference time-domain (FDTD) methods were used to investigate differences in the extraction efficiency between these samples. We took randomly distributed dipoles with unpolarized emissions at 400–550 nm in the region of MQWs while considering the EL spectra of the samples (Supplementary Section VI); the detection wavelength of the dipole (*λ*_det._) was also set to this range. Emissions from MQW were only detected in the upward direction, and the position of the monitor was above half of the wavelengths from the structure to exclude the near-field electric field. Figure 4a and b shows the schematic diagrams and simulation results for A-LED and AS-LED. We simulated additional structures to compare the effects of the air void and the SiO_2_ layer in samples with different structures besides P-LED, A-LED, and AS-LED. The size, height, and angle of the structures were confirmed by TEM results, as shown in Fig. 2d–g. The LEE values of the A-LED and AS-LED were enhanced 2.7 and 3.8 times, respectively, compared to that of the P-LED, which was attributed to substantial reduction of the total internal reflection by insertion of the air void and SiO_2_ layer. The enhancement of LEE for AS-LED was similar to the values reported for samples using patterned sapphire substrates^26, 27^. With further optimization of the structure in terms of air void size and SiO_2_ layer thickness, we expect that the extraction efficiency can be further improved.

### Characterization of electroluminescence

**Figure 5:**
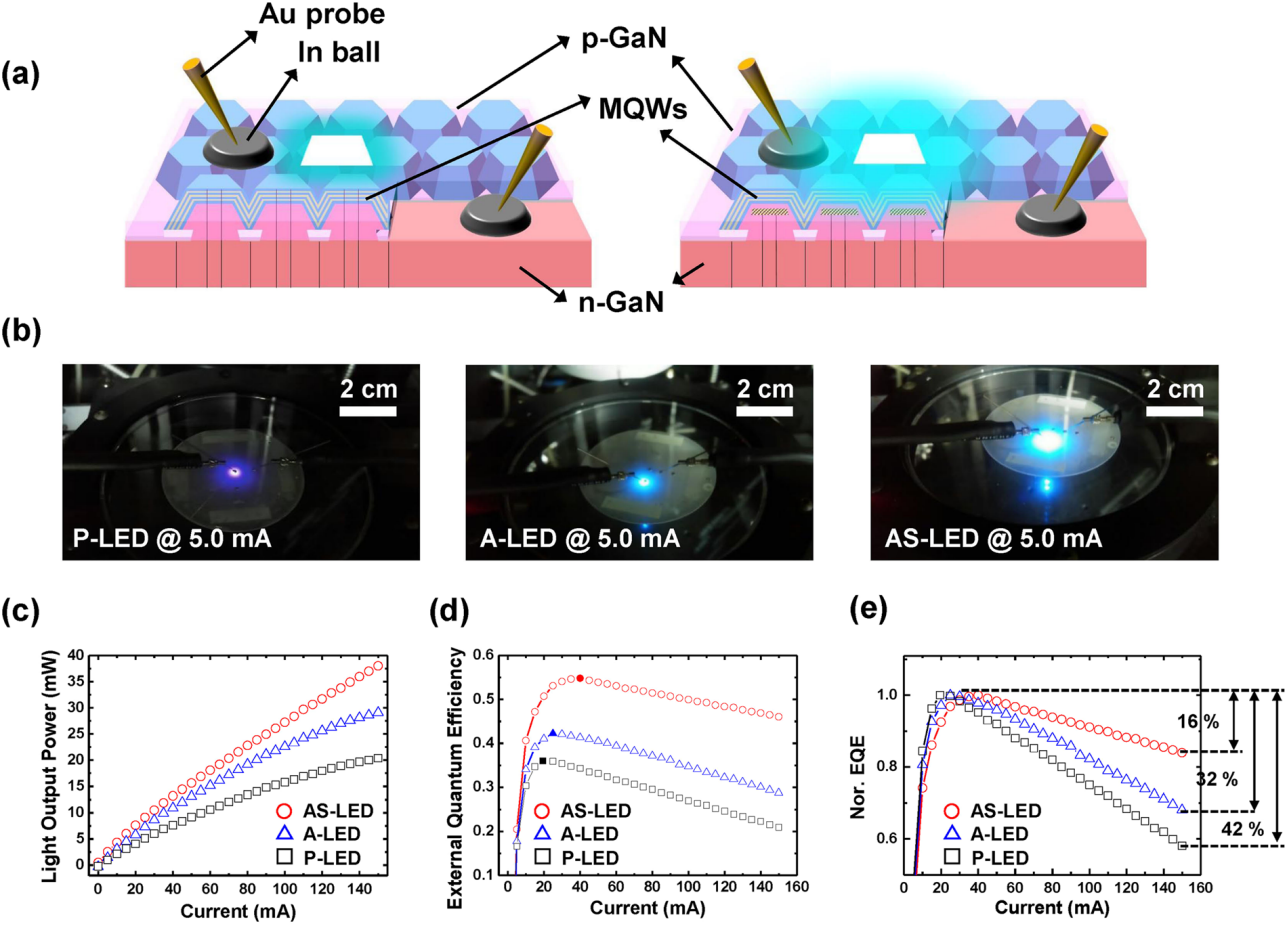
Comparison between efficiency and electrical characteristics: (**a**) Schematic layer structure of A-LED and AS-LED. (**b**) Light emission images at the same injection current (5 mA), (**c**) *L*-*I* curves, and (**d**,**e**) EQE and normalized EQE for P-LED, A-LED, and AS-LED.

Figure 5 details the efficiency and electrical characteristics of the samples. Figure 5a shows the schematic layer structure for A-LED and AS-LED. The In balls were directly attached to the wafer to serve as the *n*-type and *p*-type contact electrodes. Figure 5b shows the emission for samples at the same experiment set-up. In Fig. 5b, the scale of images is the same. As shown in Fig. 5b, we observed that the emission of AS-LED was the brightest among these samples at the same injection current (5 mA). In addition, the light output power (*L*)-current (*I*) characteristics were measured by using an integrating sphere. As shown in Fig. 5b, the light output power of AS-LED is approximately 1.3 and 1.9 times higher than those of A-LED and P-LED, respectively, at the maximum injection current (150 mA). Figure 5c and d shows the EQE and the normalized EQE at various injection currents for P-LED, A-LED, and AS-LED. In Fig. 5c, the maximum values of EQE (EQE_max_) for P-LED, A-LED, and AS-LED are estimated to be 36.0% at 20 mA, 42.2% at 25 mA, and 54.8% at 40 mA, respectively (closed symbols), and the overall efficiency of AS-LED was much higher than those of both A-LED and P-LED. In addition, we investigated the efficiency droop from normalized EQE, as shown in Fig. 5d. The efficiency droop values of P-LED, A-LED, and AS-LED, calculated as (*EQE*_peak_–*EQE*_150mA_)/*EQE*_peak_, were 42%, 32%, and 16%, respectively, the efficiency droop values of the A-LED and AS-LED were calculated at the blue emission region related to *c*-plane emission in order to compare with P-LED. In the AS-LED, we found that both the droop-onset injection current and the overall EQE are much larger, and the efficiency droop becomes less severe compared to that of the other samples^28^.

## Conclusions

We proposed an effective method for both improving the quantum efficiency and reducing the efficiency droop in GaN-based light emitting diodes, achieved by using a highly efficient 3D truncated pyramidal structure fabricated through self-aligned twofold epitaxial lateral overgrowth. This sample was compared with samples having only air voids and conventional planar structures. Compared to the P-LED and A-LED samples, the improved quantum efficiency and significant reduction of the efficiency droop in AS-LED were attributed to (i) the reduction of leakage current due to improved material quality within the QWs and an effective suppression of TDs (Fig. 2), (ii) a significant suppression of non-radiative recombination processes such as Auger and/or carrier overflow at high carrier densities by reduction of effective carrier density within the QWs (Fig. 3), and (iii) a substantial decrease in the total internal reflection by insertion of an air void and the SiO_2_ layer (Fig. 4). In addition, the emission wavelength of AS-LED was much longer than those of A-LED and P-LED, despite utilizing the same growth conditions for all samples. We believe that this result was related to the increased lattice pulling effect due to stress relaxation (Supplementary Section V)^29^. Our study was focused on the blue emission wavelength region. If this approach such as AS-LED is applied to the green and red emission regions which exhibits a lower efficiency than the blue emission region, we expect that it is much more important role for overcome of efficiency degradation problem.

## Methods

### MOCVD growth

Samples were loaded in the same metal organic chemical vapor deposition (MOCVD, Thomas Swan) chamber, and were grown under identical InGaN growth conditions. The regrowth of GaN on the two templates was carried out at 1175 °C and pressure of 100 Torr by using trimethylgallium (TMGa) and ammonia (NH_3_) as precursor sources with pure hydrogen (H_2_) as the carrier gas. The TMGa and NH_3_ flow rates were maintained at 110 sccm and 16000 sccm, respectively. Subsequently, the five periods of InGaN/GaN multi-quantum wells (MQWs) were formed on the regrown GaN layer. For growth of InGaN/GaN MQWs layer, timethylindium (TMI), TMGa and NH_3_ were used as the well and barrier precursor sources under pure nitrogen (N_2_) ambient. The flow rates of TMGa, TMI and NH_3_ were maintained at 3.5 sccm, 300 sccm, and 10000 sccm, respectively, during the growth of the five period InGaN (well) and GaN (barrier) at the reactor pressure of 300 Torr. The In_x_Ga_1−x_N wells were grown at temperature 830 °C and the GaN barriers were grown at temperature 930 °C. After growth of InGaN layer, the electron blocking layer and *p*-GaN were grown. Finally, the truncated pyramid structures after regrowth on two GaN pillar templates were observed. We prepared three samples, which were loaded into the same MOCVD chamber, and were grown in the same run by using the same growth conditions.

### Structural characterization

We used SEM (Hitach-S4800) and spherical aberration-corrected scanning transmission electron microscopy (JEOL-ARM200F) in order to investigate the structural properties including the surface morphology or QW thickness. Samples for the TEM measurement were prepared by focusing ion beam milling (FEI Helios Nanolab), and *ω*-rocking curve measurements were conducted by HRXRD (X’Pert-Pro MRD) in order to investigate the differences in material quality related to defects in the samples.

### Optical characterization

For the analysis of the optical properties, photoluminescence measurements were carried out using a 325 nm He-Cd laser. In addition, CL spectra and images were obtained using a monochromator and charge coupled device detector (Mono4, Gatan) attached to a field emission scanning electron microscope (XL30s, Philips). A source meter (Keithley 2400) was used for current injection. We used the integrating sphere with a fiber-coupled radiometrically calibrated spectrometer to measure the electrical and optical properties of the LEDs under current injection. Output power detection was carried out using an array charge coupled device (Hamamatsu, S7031–1006, back-thinned CCD array). FDTD simulations (Lumerical) were utilized to analyze the differences in light extraction between samples.

## Electronic supplementary material

Supplementary Information
