# Supplementary material for: Electrically driven, highly efficient three-dimensional GaN-based light emitting diodes fabricated by self-aligned twofold epitaxial lateral overgrowth
Source: Sci Rep. 2017 Aug 29;7:9663. doi: 10.1038/s41598-017-10086-7 (PMC5575063; doi:10.1038/s41598-017-10086-7)
Supplement: Supplementary file 1 — Supplementary Information [file 41598_2017_10086_MOESM1_ESM.pdf]

Supplementary Information for

Electrically driven, highly efficient three-dimensional GaN-based light emitting diodes fabricated by self-aligned twofold epitaxial lateral overgrowth

Yang-Seok Yoo, Hyun Gyu Song, Min-Ho Jang, Sang-Won Lee, and Yong-Hoon Cho*

Department of Physics, Korea Advanced Institute of Science and Technology, Daejeon 34141, Republic of Korea.

*Correspondence: Prof. Yong-Hoon Cho

Department of Physics, Korea Advanced Institute of Science and Technology, Daejeon 34141, Republic of Korea. Tel: (82) 42-350-2549, Email: yhc@kaist.ac.kr

I. Fabrication processes

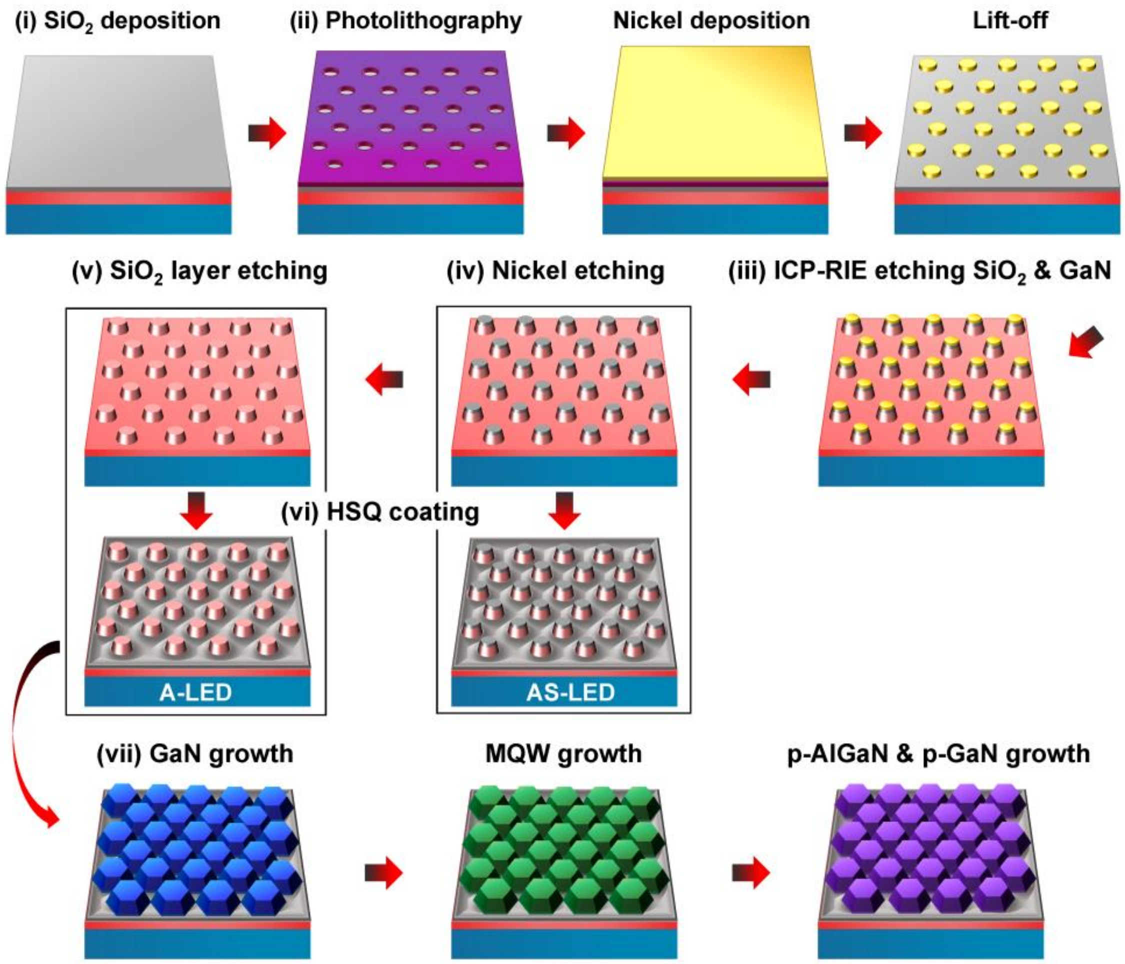

Figure S1. Schematic illumination for fabrication of 3-dimensional InGaN/GaN LED structures.

Figure S1 shows the fabrication processes for the two samples, A-LED and AS-LED. (i) The SiO_2 layer of 300 nm thickness was deposited on *n*-GaN layer grown on the sapphire substrate by plasma-enhanced chemical vapor deposition. (ii) The diameter and the center to center (*i.e.*, pitch) of circular dot with hexagonal array were designed to be in the 2 μm and 4.5 μm, respectively on the SiO_2 layer by using photolithography, and then an nickel array of 2 μm diameter was formed by a lift-off process. (iii) The SiO_2 layer was dry etched by reactive ion etching (RIE) process with CF_4(g), and the dry etching by induced coupled plasma RIE (ICP-RIE) with Cl_2(g) and Ar(g) was followed for GaN pillar structure with height of 2 μm. (iv) Samples were dipped into mixed solution of nitric acid (HNO_3) and sulfuric acid (H_2SO_4) for 5 minutes to remove the Ni disk. (v) Usually, the SiO_2 mask layer is used to suppress the plasma damage on the top side of sample in fabrication of 3D structures with nano or micro size by the top-down approach.[1] However, after GaN etching by ICP-RIE, many groups remove the SiO_2 layer before regrowth of MQWs since there is no more need. Generally, the bending of TDs were observed in low part of 3D structures made by the bottom-up approach.[2] On the other hand, it was reported that the TDs in the 3D structure fabricated by the top-down approach did not disappear.[3] Thus, we did

not remove the SiO_2 mask layer in order to block the threading dislocation induced in interface between GaN layer and sapphire substrate (AS-LED). For comparison, we fabricated a sample without the SiO_2 layer (A-LED), and additionally prepared a sample with the planar LEDs structure without the air voids and SiO_2 layer (P-LED). (vi) The hydrogen silsesquioxane (HSQ) layer was coated by using a spin coater to prevent the regrowth between GaN pillar structures during regrowth of *n*-GaN and MQWs. For curing, the temperature increases slowly at the rate of 5˚C/15 sec from 100˚C to 400˚C to avoid the crack occurring when the temperature increases fast, and the samples are baked at 400˚C for 30 min. The heigh of HSQ layer was controlled through wet etching by buffered oxide etchant solution. Finally, (vii) after *n*-GaN regrowth, we achieved the GaN structure with truncated pyramid shape from two templates, and then layers such as MQWs and *p*-GaN were regrown.

II. Cross-sectional SEM image

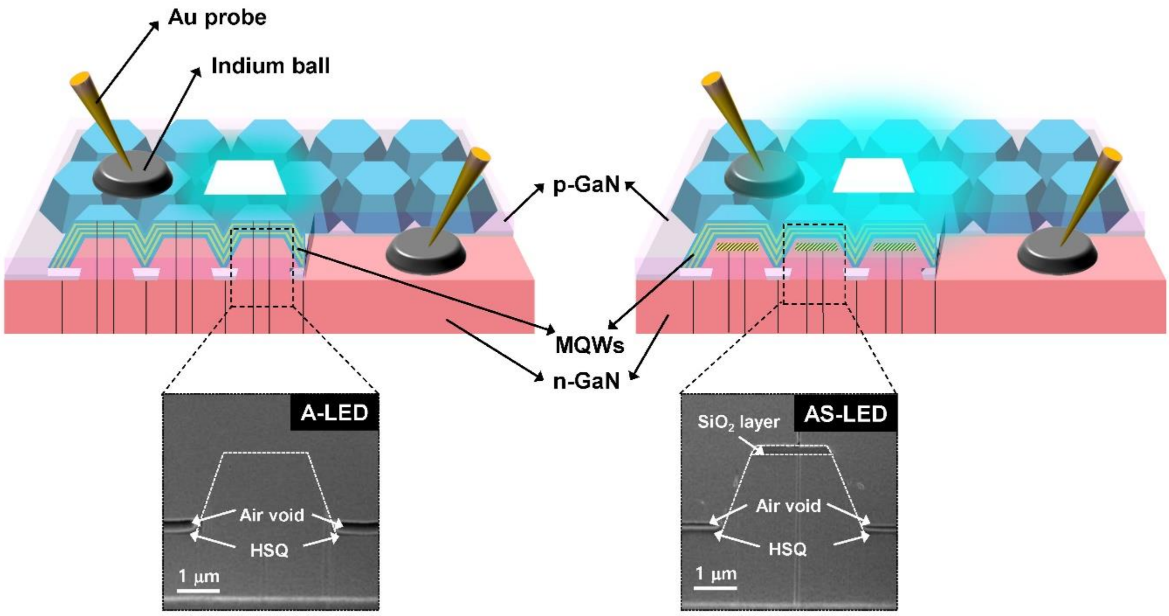

Figure S2. Investigation of difference of structural property between A-LED and AS-LED.

Figure S2 shows schematics and magnified cross-sectional SEM images of particular portion from the schematic of the A-LED and AS-LED samples, respectively. To observe the cross-section of samples, the focused ion beam technique was utilized. The white dot line in SEM images indicates GaN pillar structures shown in Fig. 1a,e before the GaN regrowth. The SiO_2 layer and the air void were clearly confirmed through cross-sectional SEM images.

III. Measurement of ω–rocking curve by using HRXRD

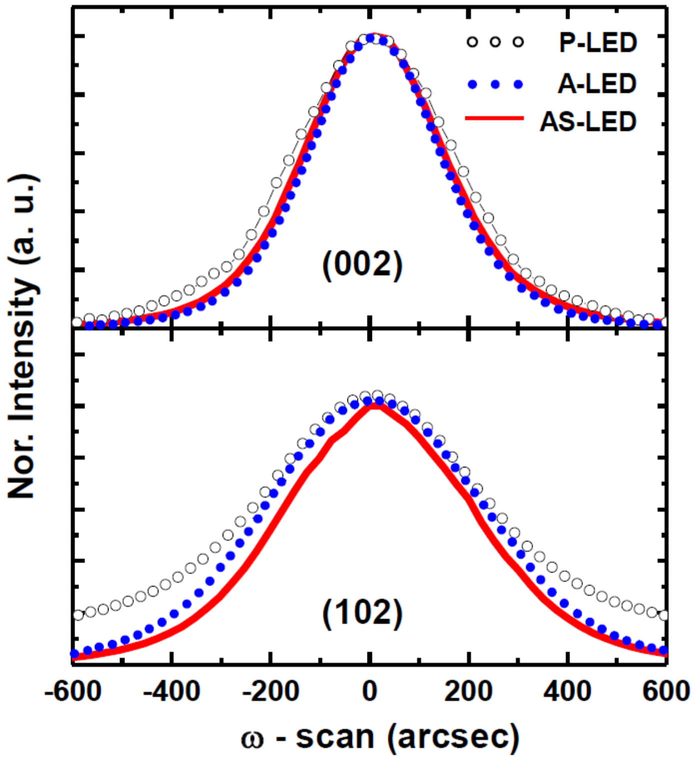

Figure S3. Symmetric (002) and asymmetric (102) reflection X-ray ω-scan rocking curves measurement for samples.

To confirm the difference of material quality for samples, we conducted ω-scan rocking curves from high resolution X-ray diffraction (HRXRD) experiments. Figure S3 shows the symmetric (002) and asymmetric (102) reflection of the HRXRD ω-scan rocking curves measured for samples. The full width at half maximum (FWHM) values of the symmetry (002) ω-scan curve were found to be 411 arcsec, 352 arcsec, and 348 arcsec, respectively, while those of the asymmetry (102) rocking curves were 609 arcsec, 527 aresec, and 474 aresec for P-LED, A-LED, and AS-LED, respectively. The FWHM of the asymmetry (102) rocking curves for the AS-LED was much decreased than that of the symmetry (002) rocking curves compared to the other samples. It has been reported that X-ray ω-scan curves on symmetric (002) planes are influenced by screw and mixed type dislocation, whereas ω-scan curves on asymmetric (102) planes are sensitive to edge type dislocation.[4] Based on these results, we confirmed that the material quality in AS-LED was improved compared to other samples due to a reduction of threading dislocations.

IV. Comparison with the leakage current at reverse bias

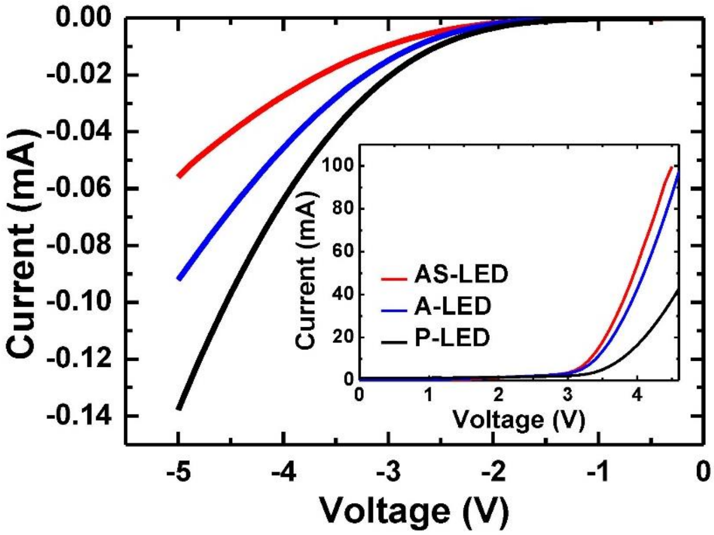

Figure S4. Leakage current with reverse bias (at -5 V) and I-V curves (inserted) measured in forward direction for P-LED. A-LED, and AS-LED, respectively.

Generally, it has been known that parasitic leakage paths along structural defects are responsible for reverse current through the carrier tunneling.[5] In Figure S4, the leakage current of AS-LED was about 5×10^{-5} A at -5 *V*, which is about 1.8 and 2.5 times smaller than those of A-LED and P-LED, respectively. Additionally, I-V curves measured in forward direction were inserted in Figure S4. We observed that the turn on voltages of A-LED and AS-LED were smaller than that of the P-LED. Although the fabrication condition for the samples was not optimized in our study, we found that the leakage current was significantly reduced in AS-LED compare to the A-LED and P-LED. These results can be attributed to the improvement of material quality by suppression of the TDs induced between the layer and the substrate by adopting SiO_2 layers and well-ordered air voids which effectively block the TD propagation to the active region.

V. Analysis of stress status of the samples by Raman measurement

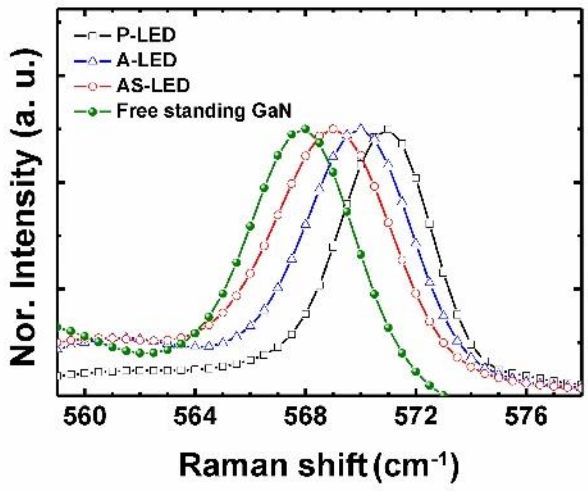

Figure S5. Micro-Raman spectra in the E_2 (high) range for P-LED (open squares), A-LED (open triangles), AS-LED (open circles), and free standing GaN (closed circles), respectively.

We conducted Raman measurement to investigate the stress status of the samples. For comparison, we additionally prepared the free standing GaN sample as a stress-free GaN reference. Figure S5 shows typical micro-Raman spectra of the GaN layer measured before the MQWs regrowth for each sample. In the micro-Raman spectra, the shift of E_2 (high) phonon mode of a GaN epilayer indicates the stress state in the epilayer.[6] The E_2 (high) phonon peaks were observed at 570.8 569.8, and 568.9 cm^{-1} for P-LED, A-LED, and AS-LED, respectively. In addition, the E_2 (high) phonon peak value of free standing GaN bulk was observed at 567.9 cm^{-1}, which is confirmed to be within the reported range (566-568 cm^{-1}) of stress-free GaN.[7] With respect to free standing GaN bulk, the frequency of E_2 (high) phonon mode shifts to a higher frequency when the layers are under compressive stress (i.e., 2.9, 1.9, and 1.0 cm^{-1} for P-LED, A-LED, and AS-LED, respectively).[8] From the results, we found that the residual compressive strain of the GaN layer for AS-LED is much smaller than those of the other samples, reflecting an efficient relaxation of the residual stress in the GaN layer by the formation of air voids and the ELOG on SiO_2 layer.[9]

VI. Analysis of emission wavelength for samples by EL spectra

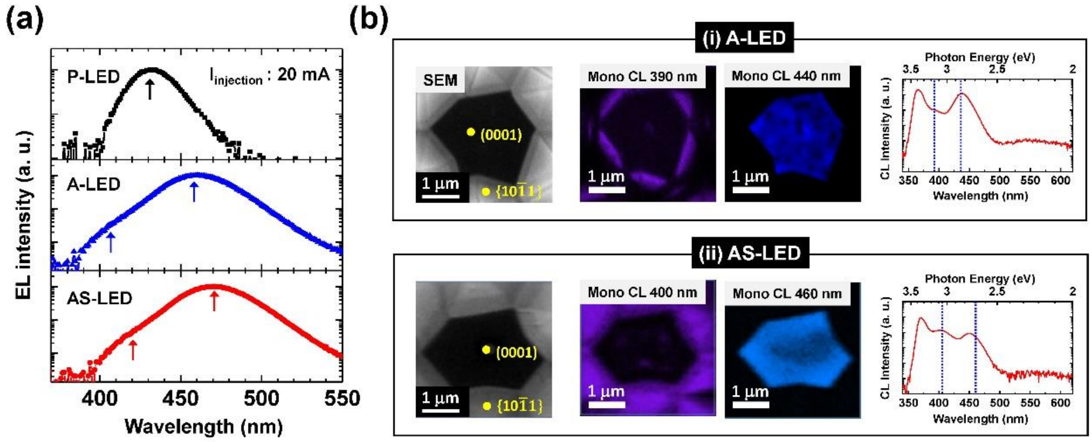

Figure S6. EL spectra and CL result (a) EL spectra at 20 mA injection current (b) Monochromatic CL images and CL spectra at acceleration voltage 5 kV.

We measured the emission peak at the injection current of 20 mA. A source meter (Keithley 2400) was used for current injection. We used the integrating sphere with fiber-coupled radiometrically calibrated spectrometer to measure the electrical and the optical properties of the LED operation under current injection. The detection of output power were performed by an array charge coupled device (Hamamatsu, S7031-1006, back-thinned CCD array). We observed that the peak wavelength between samples was different through EL spectra. The samples were fabricated with the same growth conditions. In Figure S6a, the EL peak wavelength is 431.7 nm for P-LED, 409.5 nm and 458.9 nm for A-LED, and 416.5 nm and 470.3 nm for AS-LED, respectively. Figure S6b shows the CL spectrum and images. CL experiments (Mono4, Gatan) was performed at the acceleration voltage 5 kV. The yellow points in top view SEM images for A-LED and AS-LED indicate the each facets. The one GaN band edge emission and two InGaN/GaN QW emission were observed in CL spectrum for two samples. To clarify the origin of these emission peaks, monochromatic CL mapping images were taken at wavelength of 390, 400, 440 and 460 nm. The emission of QWs on the semi-polar and *c*-plane regions was distinguished from QWs on other facets. In EL and CL experiment results, we observed that the emission wavelength for AS-LED was relatively longer than that of the other samples. Generally, the emission wavelength is attributed to the In composition and the well thickness. To analyze the difference of EL peak wavelength, we measured the well thickness from the transmission electron microscopy (TEM). As shown in Fig. 3, the well thickness in *c*-plane for samples and well thickness in semi-polar facet for A-LED and AS-LED are almost same. However, the main peak emission for AS-LED is much longer than that of the other samples. We found that the stress was much relaxed in the AS-LED with air voids and SiO_2 layer than that of P-LED and A-LED through Raman measurement. Based on our experiment results such as measurement of well thickness by TEM and comparison of the E_2 peak in Raman spectra, we thought that the large stress relaxation in AS-LED compared to the other samples lead to relatively higher In incorporation in InGaN layer. As a result, the longest emission wavelength for AS-LED was observed in EL spectra.

VII. Calculation of indium composition by TEM

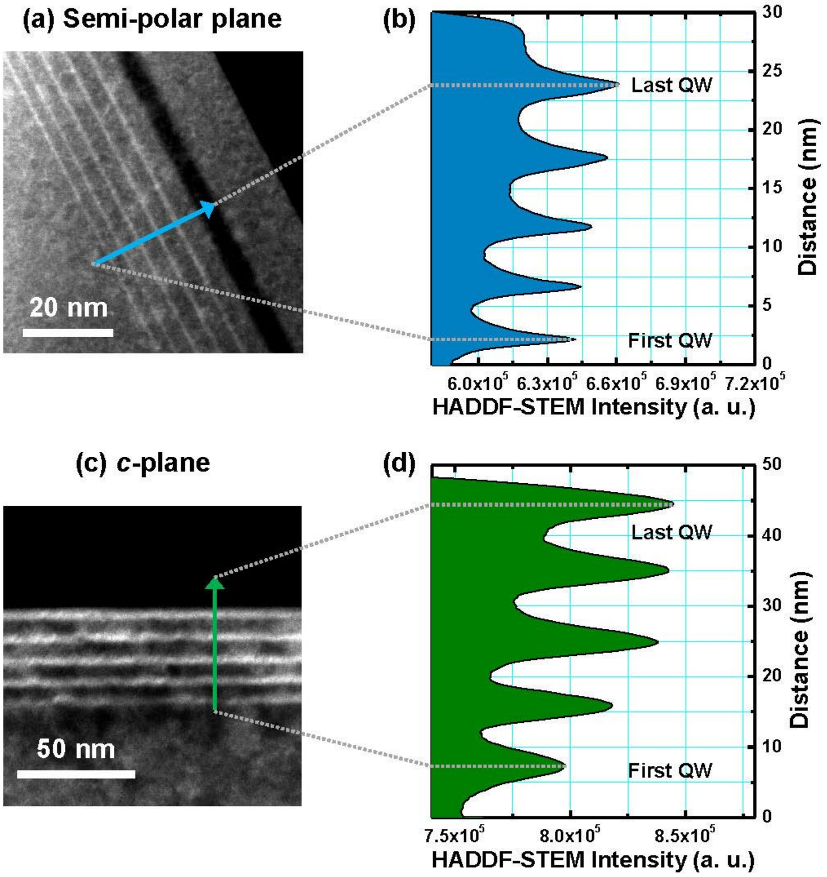

Figure S7. HAADF-STEM images (a. c) and intensity (b, d) of QWs on semi- and *c*-plane regions for AS-LED. Blue and green solid arrows indicate the scan direction.

The indium composition in QWs were calculated by using TEM results.[10] The high angle annular dark field-scanning TEM (HAADF-STEM) images of semi- and *c*-plane regions for AS-LED were shown in Figure S7. The profile of intensity on QWs for semi-and *c*-plane was extracted along the blue and green arrows direction. The indium composition inside the QWs on each plane was estimated by equation as follows:

$$I_{\mathrm{InGaN}} / I_{\mathrm{GaN}} = \frac{x\mathrm{Z}_{\mathrm{Ga}}^{\varepsilon} + (1 - x)\mathrm{Z}_{\mathrm{Ga}}^{\varepsilon} + x\mathrm{Z}_{\mathrm{N}}^{\varepsilon}}{\mathrm{Z}_{\mathrm{Ga}}^{\varepsilon} + \mathrm{Z}_{\mathrm{N}}^{\varepsilon}},$$

where I_{InGaN} and I_{GaN} are intensity of well and barrier in STEM image, x is the indium concentration, Z is the atomic number, and ε related to function of the collection angle of the HAADF-STEM detector lies typically between 1.4 and 2. This method for calculating Indium composition of QWs through STEM result is in kinematical approximation, neglecting dynamical effects, Bragg diffraction, and strain effect. The average values of the indium composition inside QWs of AS-LED (A-LED) were 0.081±0.014 (0.073±0.012) and 0.097±0.017 (0.091±0.018) for semi-polar and *c*-plane regions, respectively. This analysis result of indium composition by TEM supports well Figure S6.

VIII. Analysis for EL spectra and carrier recombination with injection current

We added the EL spectra for AS-LED with injection current. Injection currents were varied from 1 mA to 100 mA. The two peaks closed to 400 and 460 nm were observed in Figure S8. As shown in CL result, two peaks indicate the semi-polar and *c*-plane region, respectively. The In balls were directly attached to the wafer to serve as the *n*-type and *p*-type contact electrode without use of the current spreading layer such as indium thin oxide. Thus, we thought that only one peak was observed at the injection current, 1 mA (relatively low injection current) as shown in Figure S8b. With increasing injection currents from 1 to 100 mA, the emission peak of semi-polar plane was not shifted, while the emission peak of c-plane region was blue-shifted about 10 nm. The peak shift of c-plane is mainly associated with the screening effect of internal electric field.

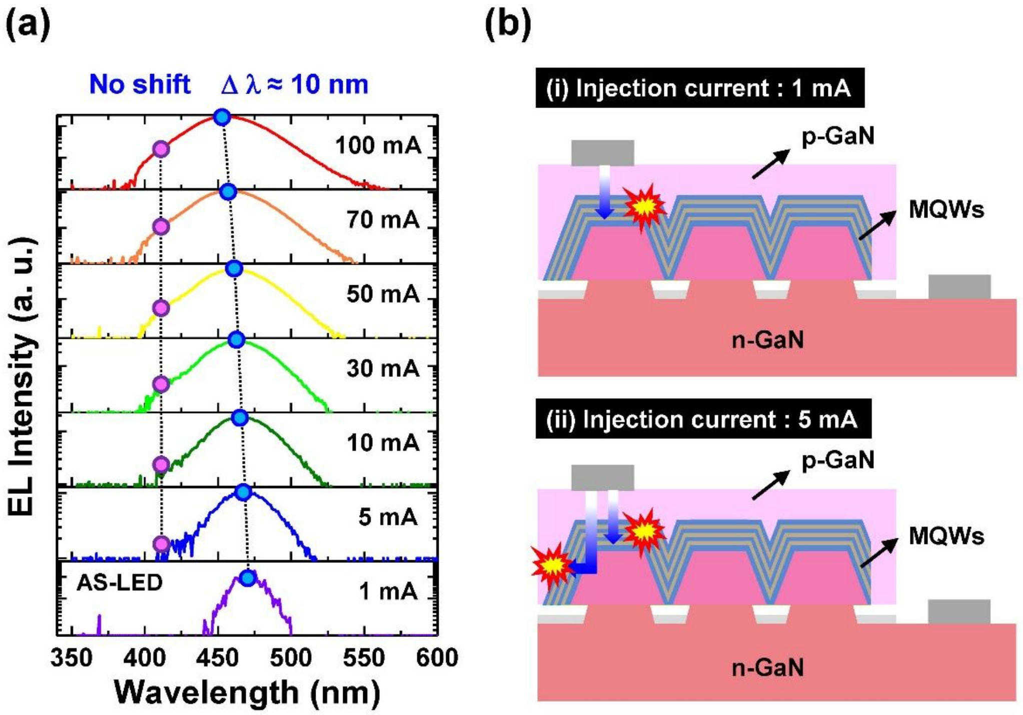

Figure S8. (a) EL spectra and (b) schematic of carrier recombination with injection current for AS-LED. The injection current is varied from 1 to 100 mA. The two peaks closed to 400 and 460 nm were observed, and indicated the emission of semi-polar and c-plane regions, respectively. With injection currents, the peak of semi-polar plane was not shifted, while the emission peak of c-plane region was blue-shifted about 10 nm.

Reference

1. Kong, D. J.; Bae, S. Y.; Kang, C. M.; Lee, D. S., InGaN/GaN microcolumn light-emitting diode arrays with sidewall metal contact. *Opt Express* **2013,** *21* (19), 22320-6.
2. Fu, B.; Cheng, Y.; Si, Z.; Wei, T.; Zeng, X.; Yuan, G.; Liu, Z.; Lu, H.; Yi, X.; Li, J.; Wang, J., Phosphor-free InGaN micro-pyramid white light emitting diodes with multilayer graphene electrode. *RSC Adv.* **2015,** *5* (122), 100646-100650.
3. Krylyuk, S.; Paramanik, D.; King, M.; Motayed, A.; Ha, J.-Y.; Bonevich, J. E.; Talin, A.; Davydov, A. V., Large-area GaN n-core/p-shell arrays fabricated using top-down etching and selective epitaxial overgrowth. *Applied Physics Letters* **2012,** *101* (24), 241119.
4. Yoo, Y.-S.; Roh, T.-M.; Na, J.-H.; Son, S. J.; Cho, Y.-H., Simple analysis method for determining internal quantum efficiency and relative recombination ratios in light emitting diodes. *Applied Physics Letters* **2013,** *102* (21), 211107.
5. Lee, S. W.; Oh, D. C.; Goto, H.; Ha, J. S.; Lee, H. J.; Hanada, T.; Cho, M. W.; Yao, T.; Hong, S. K.; Lee, H. Y.; Cho, S. R.; Choi, J. W.; Choi, J. H.; Jang, J. H.; Shin, J. E.; Lee, J. S., Origin of forward leakage current in GaN-based light-emitting devices. *Applied Physics Letters* **2006,** *89* (13), 132117.
6. Puech, P.; Demangeot, F.; Frandon, J.; Pinquier, C.; Kuball, M.; Domnich, V.; Gogotsi, Y., GaN nanoindentation: A micro-Raman spectroscopy study of local strain fields. *Journal of Applied Physics* **2004,** *96* (5), 2853-2856.
7. Kisielowski, C.; Krüger, J.; Ruvimov, S.; Suski, T.; Ager III, J.; Jones, E.; Liliental-Weber, Z.; Rubin, M.; Weber, E.; Bremser, M., Strain-related phenomena in GaN thin films. *Physical Review B* **1996,** *54* (24), 17745.
8. Hao, M.; Ishikawa, H.; Egawa, T.; Shao, C.; Jimbo, T., Anomalous compositional pulling effect in InGaN/GaN multiple quantum wells. *Applied physics letters* **2003,** *82* (26), 4702-4704.
9. Seo, T. H.; Park, A. H.; Park, S.; Kim, Y. H.; Lee, G. H.; Kim, M. J.; Jeong, M. S.; Lee, Y. H.; Hahn, Y. B.; Suh, E. K., Direct growth of GaN layer on carbon nanotube-graphene hybrid structure and its application for light emitting diodes. *Sci Rep* **2015,** *5*, 7747.
10. Amari, H.; M. Ross, I.; Wang, T.; Walther, T., Characterization of InGaN/GaN epitaxial layers by aberration corrected TEM/STEM. *Physica Status Solidi (c)* **2012,** *9*, 546-549.
